# Incidence of Benign Anal Disease After Bariatric Surgery in King Fahad Specialist Hospital, Buraydah

**DOI:** 10.7759/cureus.77409

**Published:** 2025-01-14

**Authors:** Bandar Saad Assakran, Marya A Algoblan, Majd S Alsaqabi, Amar Alsawyan, Raghad Albarrak, Yaqeen F Alrubaish, Faisal Alamer

**Affiliations:** 1 General Surgery, King Fahad Specialist Hospital, Buraydah, SAU; 2 Medical School, Qassim University, Buraydah, SAU

**Keywords:** anal disease, bariatric surgery, bariatric surgery complications, saudi arabia

## Abstract

Background: As the number of bariatric procedures rises, there is a growing need to understand the surgery's impact on other health conditions, including gastrointestinal complications. The incidence of benign anal diseases, such as hemorrhoids, anal fissures, and abscesses, has emerged as a concern among post-bariatric surgery patients. Currently, limited research exists on the incidence and risk factors associated with benign anal disease. This gap highlights the need for further research into the long-term effects of bariatric surgery on gastrointestinal health.

Objective: This study aimed to determine the incidence of benign anal diseases such as hemorrhoids, anal fissures, and anal fistulae among the patients who underwent bariatric surgery at King Fahad Specialist Hospital (KFSH) in Buraydah.

Method: This was a cross-sectional study conducted from 01/01/2021 to 31/10/2023. Data collection involved reviewing hospital records at KFSH to identify patients who had undergone bariatric surgery. After identification, these patients were contacted by phone to participate in the study. The collected data were entered into an Excel database (Microsoft Corp., Redmond, WA) and then imported into SPSS software v. 27 (IBM Corp., Armonk, NY) for statistical analysis.

Results: Among the 268 patients who underwent bariatric surgery, 255 (95.1%) had sleeve gastrectomy (SG), and 13 (4.9%) had Roux-en-Y gastric bypass (RYGB). A total of 178 patients (66.4%) reported normal bowel habits, 83 patients (31.0%) experienced constipation, and 7 patients (2.6%) reported diarrhea. Additionally, 16 (6.0%) patients developed abscesses or fistulae, and 17 (6.3%) indicated that they had noticed blood during bowel movements. Dietary assessments revealed that 123 participants (45.9%) consumed a medium-fiber diet, while 122 (45.5%) had a low-fiber diet. Changes in bowel habits were noted by 90 participants (33.6%). Furthermore, 22 patients (8.2%) reported noticing swelling around the anus during defecation.

Conclusion: After bariatric surgery, the incidence of anal disease was very low during the follow-up period. It is important to monitor bowel habits and address comorbid conditions in patients undergoing bariatric surgery to improve postoperative outcomes and quality of life. However, this study suggests that future studies consider other factors and involve a larger sample to be able to generalize the findings in a broader context.

## Introduction

Obesity has become a global health crisis over the past few decades, with rates steadily increasing worldwide. According to the World Health Organization, obesity has nearly tripled since 1975, with more than 650 million adults classified as obese in 2016 [1]. Bariatric surgery is used to treat morbid obesity and has gained prominence globally due to its effectiveness in achieving significant and sustained weight loss [2,3]. However, rapid weight loss and altered bowel habits following bariatric surgery can exacerbate the development of conditions such as hemorrhoids and anal fissures [2]. Sleeve gastrectomy is the most popular and least complex type of bariatric surgery [4]. Bariatric surgery has grown in popularity, but at the same time, it has led to a range of postoperative complications [5,6].

Gastrointestinal issues - such as acute appendicitis, acute diverticulitis, acute pancreatitis, and gallstone disease, are the most common complications [6]. There are more severe conditions, such as anastomotic leaks and bowel obstructions [7]. In this spectrum lies benign, non-life-threatening anal diseases - such as hemorrhoids, anal fissures, and abscesses - that are currently a common cause for concern after surgery [8]. Medical attention has seldom focused on the incidence of benign anal complications after bariatric surgery and thus there is limited literature on this topic [6-9].

Bariatric surgery has been associated with various postoperative complications, particularly benign anal disorders. Studies have indicated that hemorrhoids are frequently reported as the most common benign anorectal disorder following bariatric surgery [8]. Research has also identified that new-onset benign anal diseases in post-surgical patients are predominantly characterized by hemorrhoids [9]. Although the incidence of benign anal disorders after surgery has not been extensively studied, Salgado et al. noted that such conditions are observed in a notable proportion of patients during follow-ups [10].

In Saudi Arabia, bariatric surgery has become increasingly common, driven by rising obesity rates and greater access to surgical interventions. Despite this trend, there is scarce research on the incidence of post-bariatric surgery complications. A knowledge assessment study among Saudi residents in January 2024 mentioned leaking, hemorrhaging, and stomach fistulas as postbariatric surgery complications [11]. Furthermore, short-bowel syndrome has been identified as a potential complication in some patients after bariatric surgery [12]. Despite these observations, there remains a significant gap in the literature regarding the prevalence and nature of these complications, making it difficult to establish benchmarks for bariatric surgery outcomes on an international scale [12]. This study aims to fill this gap by examining the incidence of benign anal diseases following bariatric surgery at King Fahad Specialist Hospital (KFSH), Buraydah.

## Materials and methods

Study design

This research utilized a cross-sectional design and was carried out from 01/01/2021 to 31/10/2023 at KFSH in Buraydah, Saudi Arabia. This study involved all patients aged 18 years and above who underwent bariatric surgery and aimed to evaluate the incidence of benign anal disease after bariatric surgery in KFSH.

Inclusion and exclusion criteria

The study included all adult patients who underwent bariatric surgery at KFSH from January 1, 2021, to October 31, 2023. Exclusion criteria included patients with a pre-existing anal condition prior to undergoing bariatric surgery.

Sampling technique

This study employed a convenient sampling technique by leveraging the available resources and existing patient population at KFSH because it was efficient and cost-effective.

Sample size

To determine the sample size, we used the Cochrane sample size approach, which is given below:

$n = Z2(1 &minus; p)/d2$

where n is the sample size, Z is the critical statistic for a 95% confidence interval, assuming an anticipated incidence of 50% and d is the margin of error (set at 6%). The minimum acceptable sample size calculated was 266; however, we chose to use a slightly larger sample size of 268 respondents.

Data collection tools and procedures

The questionnaire was developed from recent literature in alignment with the study objective. Data collection involved identifying patients who underwent bariatric surgery by reviewing hospital files. Once identified, these patients were contacted via phone. During the call, they were asked a series of questions from a structured questionnaire designed to gather relevant information about their post-surgery outcomes and experiences. This method ensured that the data were collected directly from the patients, allowing for accurate and comprehensive responses. The information collected included demographic data (e.g., pre- and post-operation BMI), types of bariatric surgery performed, the presence of chronic diseases (e.g., hypertension, diabetes, cancer, asthma, hypothyroidism, and heart disease), bowel habits and dietary intake and symptoms and family history.

Data analysis

The data gathered from both interviews and medical records were first entered into an Excel spreadsheet (Microsoft Inc., Redmond, WA) for cleaning. Subsequently, the data were coded and transferred into SPSS software v. 27 (IBM Corp., Armonk, NY). Categorical variables were presented as counts and frequencies, while continuous variables were presented as the means and standard deviations. Inferential statistics, such as the chi-square test, were used to determine associations between categorical variables, with a predetermined significance level set at p<0.05.

Ethical approval and patient confidentiality

The research was conducted after obtaining the required approvals from the Al-Qassim Research Ethics Committee (QERC), Qassim province (H-04-Q-001). The data was collected anonymously without any personal identifiers. Therefore, it is not possible to link the data collected and analyzed as well as the results of our study with personal identities.

Once we collected the data, we applied the data to the inclusion and exclusion criteria. Then, the data was entered into an Excel format file. Informed consent was obtained from all the patients or their families.

## Results

The study included variables like preoperation BMI, post-operation BMI, and types of surgery. Table 1 shows that the mean of pre-operation BMI was 42.93 with a standard deviation of 7.07 while the mean of the post-operation BMI was 32.47 with a standard deviation of 6.37. Moreover, the majority of the participants (n=255; 95.1%) had sleeve gastrectomy surgery while 13 (4.9%) had Roux-en-Y gastric bypass surgery. In terms of comorbidities, 41 (15.3%) had cancer, asthma, hypothyroidism, and heart disease, 23 (8.6%) had diabetes and 19 (7.1%) had hypertension. Lastly, 46 (17.2%) of the participants were smokers.

**Table 1:** Demographic Information of the Participants (n=268)

Variable	Category	n (%)
Pre-operation BMI	Mean±SD	42.93±7.07
Post-operation BMI	Mean±SD	32.47±6.37
Types of Surgery	Sleeve gastrectomy	255(95.1%)
	Roux-en-Y gastric by pass	13 (4.9%)
Do you suffer from chronic diseases?	HTN	19 (7.1%)
	Diabetes	23 (8.6%)
	Others (cancer, asthma, hypothyroidism, and heart disease)	41 (15.3%)
	No	185 (69.0%)
Do you smoke	Yes	46 (17.2%)
	No	222 (82.8%)

Table 2 shows that most of the participants (n=178; 66.4%) had normal bowel habits, 83 (31.0%) had constipation, and 7 (2.6%) had diarrhea. In addition, 123 (45.9%) and 122 (45.5%) indicated that they had medium and low-fiber diets, respectively. Moreover, 90 (33.6%) participants indicated to have had a change in their bowel habits, 17 (6.3%) indicated to have had noticed blood on their toilet paper, dripping into the toilet or mixed into their stools, 64 (23.9%) of the participants their family had hemorrhoids or cancer of the colon, rectum or anus. More so, 22 (8.2%) had noticed swelling coming out of the anus during defecation, three (1.1%) had visited the ER to reduce hemorrhoids, and 36 (13.4%) underwent colonoscopy.

**Table 2:** Distribution of Bowel Habits, Dietary Fiber Intake, and Colorectal Symptoms Among Participants (N= 268)

Variable	Category	n(%)
What are your typical bowel habits?	Constipation	83 (31.0%)
	diarrhea	7 (2.6%)
	Normal	178 (66.4%)
How much fiber does your diet contain?	High-fiber diet	23 (8.6%)
	Medium-fiber diet	123 (45.9%)
	Low-fiber diet	122 (45.5%)
Have you had a change in your bowel habits?	Yes	90 (33.6%)
	No	178 (66.4%)
Has anyone in your family had hemorrhoids or cancer of the colon, rectum or anus?	Yes	64 (23.9%)
	No	204 (76.1%)
Did you notice any swelling coming out of your anus during defecation?	Yes	22 (8.2%)
	No	246 (91.8%)
Did you visit the ER to reduce hemorrhoids?	Yes	3 (1.1%)
	No	265 (98.9%)
Did you undergo colonoscopy?	Yes	36 (13.4%)
	No	232 (86.6%)

Table 3 shows that 44 (16.4%) have had chronic constipation, 40 (14.9%) have had pain during defecation and 22 (8.2%) have had pain after defecation that lasted up to several hours after defecation. Additionally, 17 (6.3%) had noticed bright blood on the stool or toilet paper after defecation, 35 (13.1%) had felt small lump or skin near the anal opening, 48 (17.9%) of the women had a vaginal childbirth and 15 (5.6%) were diagnosed with Crohn's disease.

**Table 3:** Prevalence of Constipation, Pain, and Other Colorectal Symptoms Among Participants (N= 268)

Variable	Category	n(%)
Do you have chronic constipation?	Yes	44 (16.4%)
	No	224 (83.6%)
Do you have any pain during defecation?	Yes	40 (14.9%)
	No	228 (85.1%)
Do you have pain after defecation that lasts up to several hours after defecation?	Yes	22 (8.2%)
	No	246 (91.8%)
Did you notice bright blood on the stool or toilet paper after defecation?	Yes	17 (6.3%)
	No	251 (93.7%)
Did you feel a small lump or skin near the anal opening?	Yes	35 (13.1%)
	No	233 (86.9%)
If you are a woman, did you have a vaginal childbirth?	Yes	48 (17.9%)
	No	220 (82.1%)
If you have any of those symptoms (swelling, fever, redness, discharge) from the anus is it related to any surgeries or procedures that have been done lately?	Yes	3 (1.1%)
	No	265 (98.9%)
Have you noticed blood, mucous, or pus when you defecate?	Yes	16 (6.0%)
	No	252 (94.0%)
Do you have a constant pain in the anal region that worsened when you sit down, move around or defecate or cough?	Yes	25 (9.3%)
	No	243 (90.7%)
Do you have any difficulty in controlling bowel movements?	Yes	23 (8.6%)
	No	245 (91.4%)
Did you notice any smelly discharge near the anal region?	Yes	8 (3.0%)
	No	260 (97.0%)
Do you notice any change in the skin around the anus like redness or swelling?	Yes	8 (3.0%)
	No	260 (97.0%)

Table 4 shows that 15 (5.6%) of the participants had inflammatory bowel diseases such as Crohn's disease or ulcerative colitis, three (1.1%) had trauma to the anal region, four (1.5%) had received radiation therapy to the anal region and 20 (7.5%) had a systemic disease such as diabetes, tuberculosis or HIV. Nevertheless, 16 (6.0%) had noticed blood, mucous, or pus during defecation, eight (3.0%) had noticed changes in the skin around the anus like redness or swelling, and 25 (9.3%) had constant pain in the anal region that may be worsened after sitting, moving around or defecating or coughing. Finally, 23 (8.6%) had difficulty controlling bowel movements, and three (1.1%) experienced swelling, fever, redness, and discharge from the anus related to recent surgeries or procedures.

**Table 4:** Prevalence of Inflammatory, Traumatic, and Systemic Conditions (N= 268)

Variable	Category	n (%)
Have you been diagnosed with Crohn's disease?	Yes	15 (5.6%)
	No	253 (94.4%)
Have you ever had a trauma to the anal region?	Yes	3 (1.1%)
	No	265 (98.9%)
Have you ever received a radiation therapy to the anal region?	Yes	4 (1.5%)
	No	264 (98.5%)
Do you have any systemic diseases such as diabetes, tuberculosis, or HIV?	Yes	20 (7.5%)
	No	248 (92.5%)
Do you have recurrent perianal abscesses?	Yes	16 (6.0%)
	No	252 (94.0%)

Table 5 shows that there is no statistically significant difference between incidences of benign anal disease and social demographic characteristics of the participants (P>0.05).

**Table 5:** Correlation Between Incidence of Benign Anal Disease and Social Demographic Characteristics (N= 268).

Variable	Coefficients	P-values
Pre-operation BMI	-0.006	0.927*
Post-operation BMI	-0.083	0.219
Types of surgery	0.023	0.707
Smoking	-0.048	0.430

## Discussion

The aim of this study was to determine the incidence of benign anal disease among patients who underwent bariatric surgery at King Fahad Specialist Hospital in Buraydah. The findings indicate that a significant majority of participants (n=255; 95.1%) underwent sleeve gastrectomy, while 13 (4.9%) had Roux-en-Y gastric bypass surgery. This distribution aligns with trends observed in the literature, where sleeve gastrectomy remains the most common bariatric surgical procedure due to its effectiveness and lower complication rates [13,14].

The presence of comorbid conditions underscores the importance of careful preoperative assessment and tailored postoperative care to address any emerging issues. In our study, the majority of participants had diabetes (23, 8.6%), followed by hypertension (n=19, 7.1%). Additionally, a significant proportion reported other chronic conditions, including cancer, asthma, hypothyroidism, and heart disease (n=41, 15.3%). Consistent with our findings, Salgado-Nesme et al. also reported that a substantial number of patients undergoing bariatric surgery had diabetes [10].

Benign anal diseases, such as hemorrhoids, anal fissures, and abscesses, are increasingly recognized as potential complications following bariatric surgery [9]. Our study revealed that constipation or diarrhea was observed in 31% and 2.6% of patients, respectively. Rapid weight loss, combined with altered fiber intake (where 91.4% of patients reported medium to low fiber consumption), can exacerbate conditions like hemorrhoids due to increased straining during bowel movements. A small percentage of patients (6.3%) reported the presence of blood on toilet paper post-defecation, a common sign of benign anal disease. Additionally, abscesses or fistulas were reported in 6% of patients, reinforcing the need for postoperative monitoring. The incidence of these complications aligns with findings from other studies, such as Cano-Valderrama et al., who also identified hemorrhoids as a frequent complication after bariatric surgery [9]. Moreover, Clapp et al. found in their study that the majority of the participants who received bariatric surgery were revealed to have changes in their bowel habits [14].

According to the study findings, 44 (16.4%) of the participants had chronic constipation, 40 (14.9%) had pain during defecation, and 22 (8.2%) had pain after defecation that lasted for several hours after defecation. Additionally, 17 (6.3%) noticed bright blood on the stool or toilet paper after defecation, 35 (13.1%) had felt a small lump or skin near the anal opening, 48 (17.9%) of the women had a vaginal childbirth and 15 (5.6%) were diagnosed with Crohn's disease. This was in line with a study by Bastos et al. where a small proportion of the participants found bright blood in their stool after defecation [15]. In support of this finding, a study by Allin et al. revealed that a small proportion of the participants experienced pain that lasted for some hours after defecation [16].

Nevertheless, the finding of the study revealed that 15 (5.6%) of the participants had an inflammatory bowel disease such as Crohn's disease or ulcerative colitis, three (1.1%) had a trauma to the anal region while four (1.5%) had received radiation therapy to the anal region. In addition, the study found that 20 (7.5%) had a systemic disease such as diabetes, tuberculosis, or HIV. Moreover, 16 (6.0%) noticed blood, mucous, or pus when they defecated, 8 (3.0%) noticed changes in the skin around the anus like redness or swelling, and 25 (9.3%) had constant pain in the anal region that worsened when they sat down, moved around, coughed or defecated. Among the participants, 23 (8.6%) reported experiencing difficulty in controlling bowel movements, which included issues such as involuntary leakage, straining, and irregularity. Additionally, three (1.1%) participants experienced symptoms of swelling, fever, redness, and discharge from the anus, which were related to recent surgeries or procedures. This was in line with a study by Goel et al., which noted that a small number of the evaluated patients who underwent bariatric surgery were revealed to have blood, mucous, or pus when they defecated [17]. Further, the finding in a study by Rossoni et al. adheres to this finding in that it found that a few participants from the surveyed patients shown to have constant pain in the anal region that worsened when they sat down, moved around, defecated, or coughed [18].

Future research is advised in light of these limitations. The use of cross-sectional design limits the ability to establish causation, as it captures data at only one point in time, which may not fully represent the progression or fluctuation of symptoms over time. Secondly, the reliance on telephone interviews for data collection, while practical for reaching a dispersed patient population, may lead to reporting biases, as it depends on participants' recall and self-assessment rather than objective clinical evaluations. Furthermore, the reliability of the study findings may be impacted by the fact that the survey results were based on the recorded patient data which may not reflect their present state.

## Conclusions

The findings indicate that the majority of patients had sleeve gastrectomy surgery, with a notable proportion experiencing normal bowel habits post-surgery, although some reported constipation and other colorectal symptoms. Comorbidities such as Crohn's disease, diabetes, and other systemic diseases were present in a minority of participants. The study also highlights the occurrence of symptoms like pain during and after defecation, the presence of blood, and difficulties in controlling bowel movements. Patients' lifestyles and bowel habits improved after bariatric surgery. For those with fewer comorbidities, the surgery also resulted in positive medical and psychosocial outcomes. However, only a few patients experienced complications. Therefore, future research should focus on evaluating patients with and without comorbidities separately to determine whether these complications are directly associated with the surgery.
